# Social and Demographic Factors Associated with Morbidities in Young Children in Egypt: A Bayesian Geo-Additive Semi-Parametric Multinomial Model

**DOI:** 10.1371/journal.pone.0159173

**Published:** 2016-07-21

**Authors:** Khaled Khatab, Oyelola Adegboye, Taofeeq Ibn Mohammed

**Affiliations:** 1 Centre of Health and Social Care Research, Faculty of Health and Wellbeing, Sheffield Hallam University, Sheffield S10 2BP, United Kingdom; 2 Department of Mathematics, Statistics & Physics, College of Arts & Sciences, Qatar University, Doha 2713, Qatar; 3 Centre for Energy, Environment and Sustainability, University of Sheffield, Sheffield, S10 1FL, United Kingdom; Oswaldo Cruz Foundation, BRAZIL

## Abstract

**Background:**

Globally, the burden of mortality in children, especially in poor developing countries, is alarming and has precipitated concern and calls for concerted efforts in combating such health problems. Examples of diseases that contribute to this burden of mortality include diarrhoea, cough, fever, and the overlap between these illnesses, causing childhood morbidity and mortality.

**Methods:**

To gain insight into these health issues, we employed the 2008 Demographic and Health Survey Data of Egypt, which recorded details from 10,872 children under five. This data focused on the demographic and socio-economic characteristics of household members. We applied a Bayesian multinomial model to assess the area-specific spatial effects and risk factors of co-morbidity of fever, diarrhoea and cough for children under the age of five.

**Results:**

The results showed that children under 20 months of age were more likely to have the three diseases (OR: 6.8; 95% CI: 4.6–10.2) than children between 20 and 40 months (OR: 2.14; 95% CI: 1.38–3.3). In multivariate Bayesian geo-additive models, the children of mothers who were over 20 years of age were more likely to have only cough (OR: 1.2; 95% CI: 0.9–1.5) and only fever (OR: 1.2; 95% CI: 0.91–1.51) compared with their counterparts. Spatial results showed that the North-eastern region of Egypt has a higher incidence than most of other regions.

**Conclusions:**

This study showed geographic patterns of Egyptian governorates in the combined prevalence of morbidity among Egyptian children. It is obvious that the Nile Delta, Upper Egypt, and south-eastern Egypt have high rates of diseases and are more affected. Therefore, more attention is needed in these areas.

## Introduction

Across the globe, the burden of mortality in children in poor developing countries has long constituted a big health concern. According to a UNICEF report in 2014, nearly 7 million children under five die every year, which is down from over 12 million in 1990. Most of these children (70%) are from developing countries [1]. This has prompted medical researchers and statisticians to gain insight into this health problem with the view to developing strategies to combat it. Prevalent among the diseases that constitute this burden of childhood morbidity and mortality are diarrhoea, cough and fever. The mapping of variation in risk of child morbidity can help improve the targeting of scarce resources for public health interventions. However, direct mapping of relevant environmental risk factors is difficult and this has led to investigations of environmental proxies. The current study focuses on Egypt based on the fact that the prevalence of childhood morbidity in this country has remained high during the last few years. This is due to the lack of adequate health care facilities, distribution inequality in terms of income and access to the basic essentials of life, and poverty. In 2014, the Central Agency for Public Mobilisation and Statistics reported that Egypt has a population of about 87 million people, and was regarded as the most populous country in the North Africa and Middle East region [2].

A number of indicators have been employed to measure the well-being of the people in a given country. For instance, the poverty index measures severe health deprivation by the proportion of people who are not expected to survive to age 40. Based on this metric, the 2004 Human Development Report (HDR) submitted that 3.1% (2.2 million people) of the total population of Egypt lives on less than $1 per day [3]. If the analysis were based on $2 per day, then the measure of poverty rate increases to 43.9% (30.9 million people). An important observation made by the HDR report related to inequality in income distribution, where a few (20%) rich individuals across the entire population benefit from 43.6% of the national income, while 20% of the poorest people share only 8.6%. The 2009 Human Development Report (HDR) uses the Human Poverty Index which gives a measure of overall poverty using several variables, including health deprivation, the adult illiteracy rate, the percentage of individuals without access to health services or potable water, and the percentage of children who are severely underweight; on this measure it ranks Egypt 82^nd^ among the 135 countries it includes [4,5].

The reported poverty level and income distribution inequality make the healthcare problems in Egypt particularly acute. For instance, the United Nations Children’s Fund, UNICEF (2008) reported that the child-mortality rate in Egypt was 68 deaths per 1000 children [6].

Similarly, the WHO Global Database on Anaemia (2008) concluded that more than half of school children suffer from anaemia and the prevalence of childhood stunting decreased from 39.7% in 1990 to 26.7% in 2010 worldwide [7]. Furthermore, the Egyptian Demographic and Health Survey (EDHS) showed that among children under the age of five, 9% were reported by their mothers to have suffered from diarrhoea during the two-week period before the EDHS interview [8, 9]. Children in the age range of 6–11 months were more likely to have suffered from diarrhoea than older children.

Previous studies [10, 11] which have focused on childhood disease in developing counties have typically neglected aspects of the associations between fever, diarrhoea, and cough. Notable exception to this are studies conducted in Nigeria that focused more on separate geoadditive probit models for cough, fever, and diarrhoea [12–14]. However, the diseases often coexist in the same eco-epidemiological settings and may share common risk factors, and morbidity and mortality may be a result of cumulative effects of different diseases. Khatab and Fharmeir [15], and Khatab, and Khatab and Kandala [16–17] considered the three types of diseases as a health status indicator for the latent variable model and Adegboye and Kotze used exploratory analysis [18]. Thus, much work remains to be done to cultivate a better accepting approach that allows us to investigate childhood comorbidities in a more accurate way.

In the light of the above, the aim of the study presented here is to investigate the associations between multiple overlap of disease outcomes (diarrhoea, cough and fever) and the socioeconomic and demographic indicators. This is because little or no research has extensively modelled multiple overlap disease outcomes between diseases. Furthermore, little is known about geographical overlaps in these illnesses. Gaining an understanding of such overlaps using advanced statistical modelling may expand our understanding of the epidemiology of the diseases which can then serve as a basis for designing efficient and cost-effective controls. To this end, the current work seeks to identify risk factors responsible for the geographical variation in morbidity at individual and community level. Obtaining the pattern of co-morbidity, combinations of overlapping illnesses reported in a child will allow us to have multi-categorical response, which can be analysed by multinomial regression models.

## Materials and Methods

### Study area and data

The analysis in this work is based on data available from the 2008 Egypt Demographic and Health Survey (EDHS) (S1 Data) conducted by Egypt’s Ministry of Health and Population, the National Population Council in collaboration with Macro International. (See Ministry of Health and Population, El-Zanaty and Associates, and ICF International for detail information about methods used in EDHS [8, 19]). To access EDHS data, the author first registered as a user of the DHS website and sent a project request including the title of the study, aims, research questions and a description of the analysis that the author proposed to perform with the data to the database manager. There was no need to have an ethical approval to access or to use the EDHS data.

The survey included questions designed to explore child survival and health, and socio–economic and environmental conditions at household level. The information was collected from women interviewees aged 15–49 years. In the survey, the health status of each interviewee’s ‘young’ children (aged < = 60 months in the 2008 survey) was assessed by asking the interviewee ‘Has your child had diarrhoea, cough and/or fever in the last 2 weeks?' Overall, data on 10,872 ‘young’ children was collected in the survey [8].

### Description of outcome variables

#### Diarrhoea

Diarrhoea is caused by varieties of micro-organisms such as viruses, bacteria and protozoans. The disease affects the health of people and causes loss of water and electrolytes which may lead to dehydration and death in some cases.

### Fever

Fever in children is mostly caused through viral infection. However, fever is less common and high fevers are unusual in young infants, and any fever should be considered a danger sign of very severe disease. The causes of fever could be an infection caused by germs (virus, parasites, or bacteria), or vaccinations or immunization shots. Sometimes children have fever for no known reason.

#### Cough

Cough and breathing difficulty are common problems in young children. Breastfed children with a cough or cold may have difficulties in feeding; however, breastfeeding could help to fight the diseases [20]. Along with diarrhoea, acute respiratory infection (ARI), particularly pneumonia, is a common cause of death among infants and young children [8].

The symptoms of diseases (diarrhoea, fever, and cough) are binary response variables (1 = Had disease vs. 0 = No). However, a multi-categorical outcome variable was created from the combinations of these diseases. It was created as follows: (1) if the child experienced all three illnesses; (2) if the child was sick due to both diarrhoea and fever; (3) if the child had both diarrhoea and cough; (4) if the child had both fever and cough; (5) if the child experienced diarrhoea only; (6) if the child experienced fever only; (7) if the child experienced cough only; and (0) if the child had no disease in the two weeks before the survey. Descriptions of outcome variables are shown in S2 Table.

#### Covariates

We considered the following socio-demographic factors as explanatory variables: child’s age, sex, body mass index (BMI), maternal age at first birth, place of residence, household size, and mother’s education. Also, the place of delivery and antenatal visits were used as institutional variables. Working status and wealth index were used as proxies for the socio-economic position of the household because EDHS does not collect information on household income and expenditure. Egypt comprises of 27 governorates, which were categorised by EDHS into 7 areas namely: Urban governorates, Lower Egypt urban, Lower Egypt rural, Upper Egypt urban, Upper Egypt rural and Frontier governorates (see S1 Table) [19]. However, in spatial analysis, we have used 27 governorates to investigate the spatial effects in the prevalence of overlap illnesses at the state level. This was achieved using a geo-additive semi-parametric multinomial model.

#### Statistical Analyses

Let *Y*_*ijk*_ and *π*_*ijk*_ be the illness status and probability of co-morbidity of illness. None of the three diseases (k = 0), had three diseases (diarrhoea, fever, cough) (k = 1), had diarrhoea and fever (k = 2), had diarrhoea and cough (k = 3), had fever and cough (k = 4), had only diarrhoea (k = 5), had only fever (k = 6), had only cough (k = 7).

We assumed that *Y*_*ijk*_ follows a multinomial distribution, i.e., *Y*_*ijk*_ ~ MN (1,*π*_*ijk*_) where *π*_*ijk*_ = (*π*_*ij*0_,*π*_*ij*1_,*π*_*ij*2_,….,*π*_*ij*7_)′. Given some categorical covariates, *Z*_*ij*_, metrical covariates, *υ*_*ik*_ and state-specific random effect, *S*_*ik*_, the probability of illness can be modelled thus:

$$P=(Y_{ijk}=k)=\frac{\exp(\eta_{ijk})}{1+\sum_{l=1}^{k}\exp(\eta_{ijl})},k=0,1,2,..,7$$

The predicator, *η*_*ijk*_ is given by *η*_*ijk*_ = *z*_*ij*_*β*_*k*_ + *f*_*k*_(*υ*_*ij*_) + *S*_*ik*_, where *η*_*ijk*_ is a known response function with a logit link function, *β*_*k*_ is the vector of the regression parameters (explanatory variables such as gender, place of residence, etc.) and *f*_*k*_ is a smooth function for the metrical covariates (child's age and maternal age at first birth) which were assumed to be nonlinear in some previous studies for each of the status categories k *[15]*. We have included these variables as nonlinear metrical covariates in the early stage of this study; however, the pattern did not show exactly the significance level of each category. Therefore, we used these covariates as linear effects instead to assess the significance level of each category (see S1 and S3 Tables). We set the first category as reference and used the logit link for modelling.

The random effects, *S*_*ik*_, are district or sub-district specific factors, and can be split into spatially structured variation (*θ*_*ik*_) and unstructured multinomial heterogeneity (*ϕ*_*ik*_), such that, *S*_*ik*_ = *θ*_*ik*_ + *ϕ*_*ik*_. P-spline priors were assigned to the functions *f*_*1*_,…,*f*_*p*_, while a Markov random field prior was used for *f (s*_*i*_*)* [21–22].

To estimate model parameters, we applied the fully integrated Bayesian approach. Though the estimation method with this model is difficult, the estimated posterior odds ratios (OR) that were produced could be understood as similar to those of normal logistic models. The analysis was carried out using version 2.1 of the BayesX software package, which certifies Bayesian inference based on Markov chain Monte Carlo (MCMC) simulation techniques [23–24].

Descriptive statistics and chi-squared tests were carried out to identify associations between predictors, confounders, and outcome variables using version 13 of STATA. P-values of less than 0.05 were considered statistically significant. The multinomial logistic regression model was used to identify associations between outcome variables (combinations of health indicators) and all predictors. Posterior Odds Ratios and their 95% confidence intervals were calculated (using BayesX) as risk estimates (OR; 95%CI) (S3 Table).

## Results

S1 Table shows the distribution of the factors that were considered in the analysis and their associations with the three indicators of the childhood morbidity.

The following factors were significantly associated with diarrhoea (S1 Table): household size (P<0.001); antenatal visit (P = 0.03); and wealth index (P = 0.006). For fever, the significant factors were place of residence (P = 0.01); antenatal visit (P = 0.02); wealth index (P = 0.002); and mother’s education (P = 0.02). Cough was significantly associated with sex of child (P = 0.04); antenatal visit (P<0.001); working status (P<0.001); and wealth index (P = 0.003). BMI was classified according to WHO guidelines [25]. Results showed that 62% of children with diarrhoea, 65% of children with fever, and 68% of children with cough had overweight or obese mothers. However, BMI has only statistical significant effect on diarrhoea (P<0.001). S1 Table also shows the prevalence of the three ailments by governorates of Egypt. It indicates that most of governorates are significantly affected by the co-morbidities. Upper Egypt (urban and rural) seems to have the highest percentages of child’s diseases compare to other governorates. It shows that 20.4% children who live in Upper Egypt urban had cough while 13.9% had diarrhoea in this governorate.

S2 Table presents the distribution of the outcome categories. It shows that, of the children who had one or more of the three diseases, most (6.84%) had fever and cough followed by those who had only diarrhoea (4.6%). Few who had diarrhoea and cough (0.59%) or diarrhoea and fever (0.79%).

S3 Table participants split within the seven categories of multi-categorical indicators of morbidities.

It presents the results of the multinomial logistic regression analysis. The table displays the estimated effects of the categorical variables: BMI, sex of child, place of residence, household size, antenatal visit, place of delivery, working status, living in an urban area, mother’s working status, wealth index, mother’s education, and Egyptian governorate on combination of diseases in Egypt. The first column presents the odds of having three diseases versus those of having none of them. The results showed that children under 20 months of age were more likely to have the three diseases (OR: 6.8; 95% CI: 4.6–10.2) than children who were between 20 and 40 months (OR: 2.14; 95% CI: 1.38–3.3). This is also true for the other different combination of diseases, particularly for the diarrhoea & fever (OR: 17.14; 95% CI: 5.3–55.2), diarrhoea & cough (OR: 4.9; 95% CI: 2.2–10.6), and those who had diarrhoea only (OR: 4.9; 95% CI: 3.6–6.5). Additionally, the likelihood of having the three diseases (OR: 1.2; 95% CI: 0.8–1.4) and having diarrhoea & cough (OR: 1.6; 95% CI: 0.96–2.7) was higher for boys than for girls. On the other hand, the association between male and the other combination (e.g. diarrhoea & cough, fever & cough, etc.) of diseases was not that high.

The children of mothers who were over 20 years of age were more likely to have only cough (OR: 1.2; 95% CI: 0.9–1.5) and only fever (OR: 1.2; 95% CI: 0.91–1.51) compared with their counterparts. There is an association between mothers who had BMI over 18 (overweight/obese) and fever only and cough only respectively. However, BMI did not show significant correlation with the rest of rest of combined morbidity compared to no illness. Likewise, children living in an urban area were more likely to have the three types of diseases (OR: 1.8; 95% CI: 1.3–2.3).

The estimated odds ratio shows that children from mothers with some antenatal visits during pregnancy has increased risk of all the seven categories of diseases except the combination of diarrhoea and cough. An increase in household size increases the chance of a child suffering fever and cough, and cough only. Our results show mixed association between place of residence and the seven categories of diseases. Additionally, children delivered at home or other places have higher likelihood of having only cough or only fever compared with children delivered at a public or private hospital.

Similarly, the chance of having the three diseases is slightly higher for children with working mothers. Maternal education is a significant risk factor for childhood morbidity; mothers with secondary and higher education have lower risk of having the three diseases, fever and cough, and diarrhoea only. Similarly, children from poorest and poorer households are 2.5 times more likely to have all the three diseases and the same goes for the remaining six categories of the disease.

**Fig 1:**
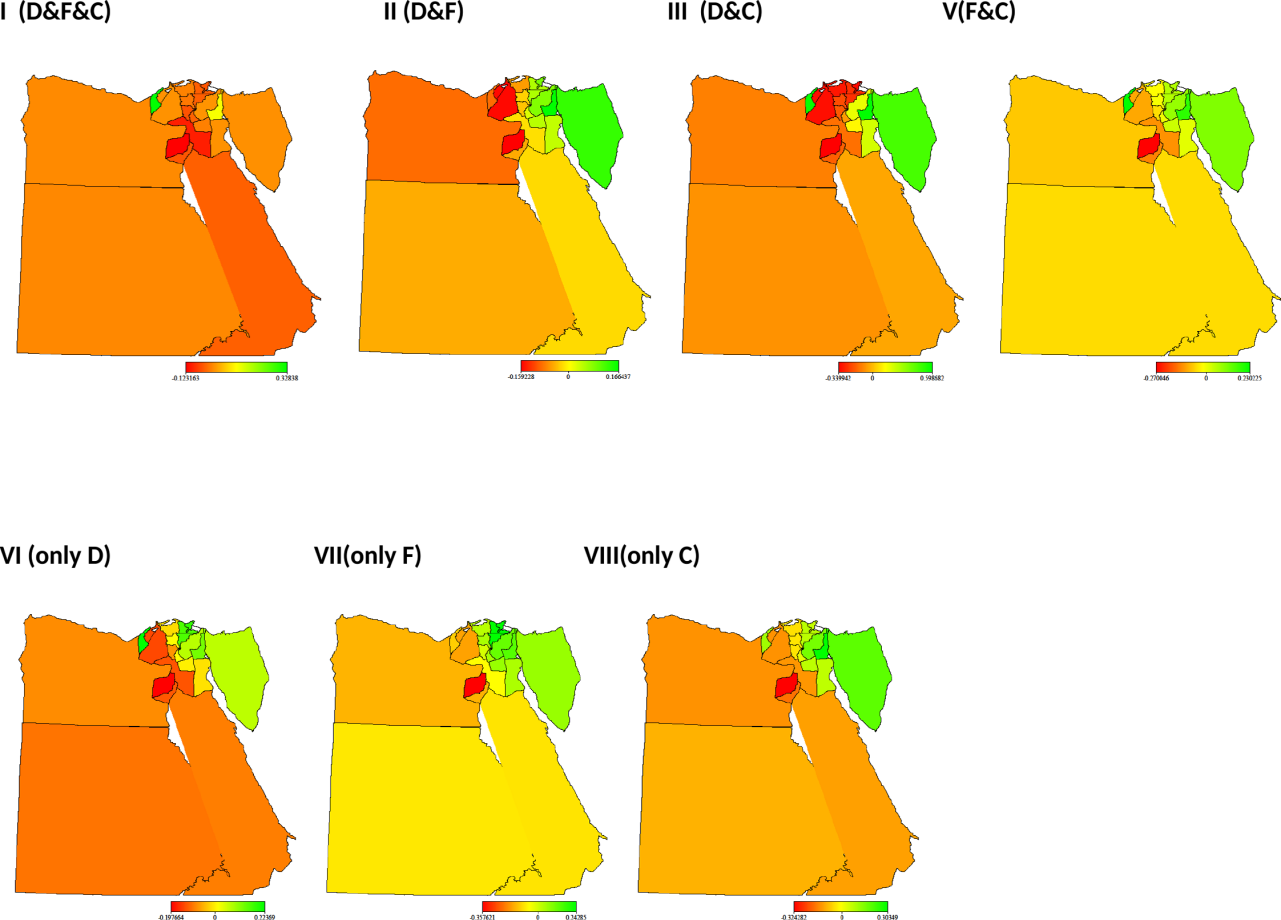
Maps of Egypt showing the spatial effects (posterior OR) on Co-morbidities: I. all illnesses vs. no illnesses, II. Diarrhoea and Fever vs. no illnesses, III. Diarrhoea and Cough vs.no illnesses, V. Fever and Cough vs.no illnesses, VI. only Diarrhoea vs.no illnesses, VII. Only Fever vs.no illnesses, VIII. Only Cough vs.no illnesses.

Fig 1 shows the structured spatial effects of co-morbidities. The results confirmed evidence of regional differences in the likelihood of a child having a combination of diseases. From the graph, it is clear the North-eastern region of Egypt has a higher likelihood than most other regions. Using Frontier governorate as a reference, children residing in Upper Egypt Rural have the highest risk of having all of three diseases, and diarrhoea and fever. Similarly, the likelihood of having cough only, diarrhoea only, and fever and cough is higher in Upper Egypt urban than any other region, while the likelihood of fever only is highest in the Urban region (S3 Table).

## Discussion

This study reiterates the importance of socio-demographic variables and their effects on morbidities. An understanding of the dynamics of comorbidities is crucial in assessing the health situation of a population. Several diseases may play different roles in the body, and thus the concept of multiple analyses is important. This study provides an important empirical study of comorbidity among children in Egypt.

We found children less than 20 months are at higher risk of any of combination of cough, fever, and diarrhoea, in single or multiple morbidities. However, the likelihood of diarrhoea and fever was higher among children of less than 20 months than any other diseases. This is not surprising because diarrhoea is very common in children [26]. Our results showed prevalence of disease to be highest among children aged 6 to 12 months of age and these results are in consistent with some previous studies (Khatab and Fahremir; Khatab; Khatab, and Kandala) [15, 17]. This result suggests that male children were more likely to have all but one of the seven categories of disease classification except diarrhoea and fever than their female counterparts. This phenomenon was attributed to biological reasons in Khatab and Fahrmeir [27].

We have found children from women above 20 years to have higher risk of the three diseases, fever only and cough only than younger women. However, children from younger woman have increase chances of diarrhoea and fever, diarrhoea and cough, fever and cough, and diarrhoea only which is consistent with previous studies [15, 28].

The odds of BMI did seem to have a slight effect on the morbidities. However, this result is not consistent with some previous studies which reported that parents with low BMI values are malnourished and are therefore likely to have undernourished and weak children [13]. Children from mothers with some antenatal visit had increased risk of most of the diseases. This seems counter-intuitive; however antenatal visits may be a sign of problem pregnancies and perception bias of morbidity [15]. The lower quintile of the wealth index represents the households with lowest socio economic status, was associated with higher risk of all categories of morbidities except fever only. This result confirms that household wealth inequality is significantly associated with childhood diseases and it suggests that reducing poverty and making services more available and accessible to the poor are essential to improving overall childhood health.

From the results, we have found that mothers’ education plays a significant role in her child’s morbidity, which may be as result of community enlightenment, communication of medical risks and increase immunisation uptake [29]. Since a lot of the diseases that affect children are vaccine-preventable, immunisation remains the most important and cost-effective public health intervention for protecting individuals, families and communities from these diseases. Such intervention options can help in truncating the cycles of disease transmission [29]. The increased odds ratio of having disease(s) was higher for a few comorbidity of working mothers. This might be because working mothers usually leave their children at home in the hands of relatives or a nanny. Often, the duration of full breastfeeding is shortened for working mothers, which may lower the chance of child’s survival [30]. Moreover, caretakers are mostly illiterate; this could have a side effect on the health of the child in the early months [27].

There was evidence of geographical variation of combinations of disease co-morbidities in Egypt. The higher likelihood of co-morbidities observed in the North-eastern part of the country was attributed to Food insecurity associated with water supplies and quality of water could be a reason for these negative effects in this area. [27]

## Conclusion

We have presented multivariate spatial models for three combined diseases in Egypt. These types of epidemiological studies have been relatively few and in some cases have been limited to exploratory analysis (e.g. Adegboye and Kotze, Kazembe, and Kandala, Kazembe *et al*. [18, 31, 32]).

We have seen significant differences in morbidity across Egypt and the role played by socio demographical variables.

This study is novel, because the dependency between multiple morbidities has not been examined before in Egypt. The findings provide the possibility of simultaneously looking into issues of multiple morbidities among children in Egypt. The maps could be used for targeting regional development in the future. It is obvious that the Nile Delta, Upper Egypt, and south-eastern Egypt have high rates of diseases and are more affected. Therefore, more attention is needed in those areas. These areas are more likely to have higher poverty compared with other areas, due to poor health facilities and the quality of health care provided for maternal and child health or even poor care and misdiagnosis during hospital care [16].

### Strength and Limitations of this Study

To our knowledge, this is the first study of its kind in Egypt that describes multiple overlap disease outcomes between diseases in Egyptian children, identifies geographical overlaps in these illnesses and sources of changes, and assesses associated factors contributing to the changes in the overlaps between the illnesses.

Although demographic and health surveys (DHS) are comparable to nationally representative household surveys that have been conducted in more than 85 countries worldwide since 1984, one of the limitations in this data is that the potential selection bias of the data since data were collected from a self-reported survey, rather than from medical examinations conducted by trained medical staff. Therefore, the responses may be subject to recall bias or misinterpretation of the children’s symptoms.

When this research project started, EDHS 2008 was the most recent dataset and this is why the authors did not consider the updated survey from 2014.

## Supporting Information

S1 Data EDHS data 2008. (SAV)

S1 Table Distribution of factors analysed in childhood morbidity in Egypt (DHS 2008) (DOCX)

S2 Table Cross-classification of children by diarrhea, fever, and cough in Egypt (DHS 2008) (DOCX)

S3 Table Association of childhood morbidity according to selected socio-demographics factors (DOCX)
