# Supplementary material for: Social and Demographic Factors Associated with Morbidities in Young Children in Egypt: A Bayesian Geo-Additive Semi-Parametric Multinomial Model
Source: PLoS One. 2016 Jul 21;11(7):e0159173. doi: 10.1371/journal.pone.0159173 (PMC4956117; doi:10.1371/journal.pone.0159173)
Supplement: S1 Table — (DOCX) [file pone.0159173.s002.docx]

**S1Table: Distribution of factors analysed in childhood morbidity in Egypt (DHS 2008)**

|  | **Diarrhea recenty (% and N)** | | | **Fever recently (% and N)** | | | **Cough recently (% and N)** | | |
| --- | --- | --- | --- | --- | --- | --- | --- | --- | --- |
| **Sex of child** | **Yes** | **No** | **P values** | **Yes** | **No** | **P values** | **Yes** | **No** | **P values** |
| Male | 520(53.1) | 4861(50.6) | 0.1 | 212(15.5) | 1156(84.5) | 0.13 | 803(53.2) | 4548(50.4) | 0.04 |
| Female | 459(46.9) | 4752(49.4) |  | 97(10.9) | 797(89.1) |  | 707(46.8) | 4499(49.6) |  |
| **Place of residence** |  |  |  |  |  |  |  |  |  |
| Urban | 380(38.8) | 3495(36.4) | 0.12 | 581(39.4) | 3294(36.1) | 0.01 | 576(38.1) | 3294(36.3) | 0.1 |
| Rural | 599(61.2) | 6118(63.6) |  | 895(60.6) | 5821(63.9) |  | 934(61.9) | 5773(63.7) |  |
| **Household size** |  |  |  |  |  |  |  |  |  |
| Small household | 172(17.8) | 1055(11.1) | 0.001 | 206(14.2) | 1021(11.3) | 0.5 | 190(12.8) | 1034(11.5) | 0.02 |
| Medium household | 346(35.9) | 3991(42) |  | 519(35.7) | 3819(42.4) |  | 566(38.2) | 3769(42.1) |  |
| Large household | 446(46.3) | 4447(46.8) |  | 728(50.1) | 4163(46.2) |  | 727(49) | 4156(46.4) |  |
| **Antenatal visit** |  |  |  |  |  |  |  |  | 0.000 |
| No | 213(21.8) | 2659(27.7) | 0.03 | 331(22.4) | 2542(27.9) | 0.02 | 306(20.3) | 2564(28.3) |  |
| Less 5 visits | 159(16.2) | 1237(12.9) |  | 911(61.8) | 1163(12.8) |  | 936(62) | 5374(59.3) |  |
| More 5 | 607(62) | 5713(59.5) |  | 233(15.8) | 5407(59.3) |  | 267(17.7) | 1126(12.4) |  |
| **Place of delivery** |  |  |  |  |  |  |  |  | 0.4 |
| Home | 255(26) | 2878(29.9) | 0.1 | 421(28.5) | 2711(29.7) | 0.7 | 2706(29.9) | 422(27.9) |  |
| Public Health | 295(30.1) | 2611(27.2) |  | 423(28.7) | 2483(27.2) |  | 2464(27.2) | 439(29.1) |  |
| Private Health | 428(43.7) | 4083(42.5) |  | 630(42.7) | 3881(42.6) |  | 3859(42.6) | 646(42.7) |  |
| other | 1(0.1) | 39(0.4) |  | 2(0.1) | 38(0.4) |  | 36(0.4) | 4(0.3) |  |
| **Working staus** |  |  | 0.2 |  |  |  |  |  | 0.001 |
| Yes | 111(12.4) | 11187(11.3) |  | 189(12.8) | 1109(12.2) | 0.5 | 1291(85.7) | 1080(11.9) |  |
| No | 868(88.7) | 8419(87.6) |  | 1286(87.2) | 8000(87.8) |  | 216(14.3) | 7983(88.1) |  |
| **Wealth index** |  |  |  |  |  |  |  |  | 0.003 |
| Poorest | 2172(22.6) | 251(25.6) | 0.006 | 399(27) | 2025(22.2) | 0.002 | 402(26.6) | 2019(22.3) |  |
| Porrer | 1969(20.5) | 204(20.8) |  | 280(19) | 1891(20.7) |  | 299(19.8) | 1868(20.6) |  |
| Middle | 2014(21) | 212(21.7) |  | 315(21.3) | 1911(21) |  | 316(20.9) | 1906(21) |  |
| Richer | 1778(18.5) | 164(16.8) |  | 243(16.5) | 1699(18.6) |  | 238(15.8) | 1702(18.8) |  |
| Richest | 1680(17.5) | 148(15.1) |  | 239(16.2) | 1589(17.4) |  | 255(16.9) | 1572(17.3) |  |
| **Mother’s Education** |  |  |  |  |  |  |  |  | 0.2 |
| No | 342(34.9) | 3339(34.7) | 0.1 | 537(36.4) | 3144(34.5) | 0.02 | 542(35.9) | 3131(34.5) |  |
| Primary | 164(16.8) | 1437(14.9) |  | 232(15.7) | 1367(15) |  | 231(15.3) | 1366(15.1) |  |
| Secondary | 377(38.5) | 3657(38) |  | 555(37.6) | 3480(38.2) |  | 564(37.4) | 3467(38.2) |  |
| Higher | 96(9.8) | 1180(12.3) |  | 152(10.3) | 1124(12.3) |  | 173(11.5) | 1103(12.2) |  |
| **Mother's age** |  |  |  |  |  |  |  |  |  |
| <= 20 years | 470(48) | 4491(46.7) | 0.2 | 693(47) | 4266(46.8) | 0.4 | 808(53.5) | 4251(46.9) | 0.4 |
| >20 years | 509(52) | 5122(53.3) |  | 783(53) | 4849(53.2) |  | 1510(14.3) | 4816(53.1) |  |
| **BMI** |  |  |  |  |  |  |  |  |  |
| Underweight (BMI < 18.5) | 10(1) | 80(0.8) | 0.001 | 15(1) | 75(0.8) | 0.05 | 75(0.8) | 15(1) | 0.19 |
| Normal weight (BMI 18.5-24.9) | 349(36.2) | 2883(30.5) |  | 487(33.6) | 2745(30.6) |  | 2735(30.7) | 491(33.1) |  |
| Overweight (BMI 25-29-29) | 385(40) | 3946(41.7) |  | 599(41.3) | 3732(41.6) |  | 3740(41.9) | 586(39.5) |  |
| Obese (BMI ≥30) | 219(22.7) | 2546(29.9) |  | 350(24.1) | 2414(29.9) |  | 2371(26.6) | 390(26.3) |  |
| **Governorate** |  |  |  |  |  |  |  |  |  |
| Urban Governorates | 130(9.5) | 1238(90.5) | 0.01 | 212(15.5) | 1156(84.5) | 0.01 | 187(13.7) | 1179(86.3) | 0.026 |
| Lower Egypt urban | 56(6.3) | 838(93.7) | 0.006 | 97(10.9) | 797(89.1) | 0.002 | 97(10.9) | 796(89.1) | 0.04 |
| Lower Egypt rural | 190(6.4) | 2763(93.6) | 0.000 | 276(9.3) | 2678(90.7) | 0.000 | 270(9.2) | 2680(90.8) | 0.000 |
| Upper Egypt urban | 171(13.9) | 1063(86.1) | 0.001 | 226(18.3) | 1008(81.7) | 0.05 | 252(20.4) | 981(79.6) | 0.000 |
| Upper Egypt Rural | 393(11.1) | 3132(88.9) | 0.09 | 594(16.9) | 2929(83.1) | 0.24 | 642(18.2) | 2878(81.8) | 0.000 |
| Frontier Governorates | 39(6.3) | 579(93.7) | 0.01 | 71(11.5) | 547(88.5) | 0.01 | 62(10.1) | 553(89.9) | 0.02 |
|  |  |  |  |  |  |  |  |  |  |
