# Supplementary material for: Social and Demographic Factors Associated with Morbidities in Young Children in Egypt: A Bayesian Geo-Additive Semi-Parametric Multinomial Model
Source: PLoS One. 2016 Jul 21;11(7):e0159173. doi: 10.1371/journal.pone.0159173 (PMC4956117; doi:10.1371/journal.pone.0159173)
Supplement: S2 Table — (DOCX) [file pone.0159173.s003.docx]

**S2 Table : Cross-classification of children by diarrhea, fever, and cough in Egypt (DHS 2008)**

| Diseases | N | % |
| --- | --- | --- |
| Only Diarrhea | 500 | 4.6 |
| Only Fever | 318 | 2.9 |
| Only Cough | 373 | 3.43 |
| Three diseases | 328 | 3 |
| Diarrhea& Fever | 86 | 0.79 |
| Diarrhea& Cough | 64 | 0.59 |
| Fever & Cough | 744 | 6.84 |
