# Supplementary material for: Social and Demographic Factors Associated with Morbidities in Young Children in Egypt: A Bayesian Geo-Additive Semi-Parametric Multinomial Model
Source: PLoS One. 2016 Jul 21;11(7):e0159173. doi: 10.1371/journal.pone.0159173 (PMC4956117; doi:10.1371/journal.pone.0159173)
Supplement: S3 Table — (DOCX) [file pone.0159173.s004.docx]

**S3 Table: Association of childhood morbidity according to selected socio-demographics factors**

|  | **Posterior OR (CI: 95%)** | | | | | | |
| --- | --- | --- | --- | --- | --- | --- | --- |
| **Assicated Factors** | **Had three diseases vs. no ilness**  **(N=328)** | **Had Diarrhea& Fever vs. no ilness**  **(N=86)** | **Had Diarrhea& Cough vs. no ilness**  **(N=64)** | **Had Fever&Cough vs. no ilness**  **(N=744)** | **Had only Diarrhea vs. no ilness**  **(N=500)** | **Had only**  **Fever vs. no ilness**  **(N=318)** | **Had only Cough vs. no ilness**  **(N=373)** |
| **Child’s age** |  |  |  |  |  |  |  |
| <= 20 month | 6.8(4.6-10.2) | 17.14(5.3-55.2) | 4.9(2.2-10.6) | 1.5(1.2-1.9) | 4.9(3.6-6.5) | 2(1.4-2.7) | 1.2(.89-1.5) |
| 20-40 month | 2.14(1.38-3.3) | 7.2(2.2-23.9) | 1.5(0.58-3.5) | 1.3(1.07-1.6) | 2.3(1.6-3.2) | 1.6(1.2-2.3) | 0.9(0.68-1.2) |
| 40-60 month | 1 | 1 | 1 | 1 | 1 | 1 | 1 |
| **Mother’s age** |  |  |  |  |  |  |  |
| <= 20 years | 1 | 1 | 1 | 1 | 1 | 1 | 1 |
| >20 years | 1.04(0.82-1.3) | 0.9(0.6-1.5) | 0.9(0.5-1.6) | 0.9(0.7-1.08) | 0.8(0.7-1.08) | 1.2(0.91-1.51) | 1.2(0.9.1.5) |
| **BMI** |  |  |  |  |  |  |  |
| Underweight <18.5 | 1 | 1 | 1 | 1 | 1 | 1 | 1 |
| Normalweight (BMI 18.5-24.9) | 1.05(0.3-3.5) | 1.15(0.14-8.29) | 1.02(0.29-4.8) | 0.57(0.3-1.2) | 0.95(0.7-1.2) | 2.4(0.3-16) | 1.4(0.35.7) |
| Overweight (BMI 25-29.9) | 0.9(0.28-3.02) | 0.44(0.05-3.37) | 0.4(0.26-1.5) | 0.54(0.27-1.06) | 0.7(0.28-1.5) | 2.9(0.4-18) | 1.3(0.3-5.1) |
| Obese (BMI ≥30) | 0.85(0.25-2.8) | 0.5(0.06-4.17) | 0.5(0.2-1.6) | 0.52(0.26-1.05) | 0.6(0.26-1.44) | 2(0.3-15) | 1.4(0.3-5.9) |
| **Sex of child** |  |  |  |  |  |  |  |
| Female | 1 | 1 | 1 | 1 | 1 | 1 | 1 |
| Male | 1.2(0.8-1.4) | 0.9(0.61-1.4) | 1.6(0.96-2.7) | 1.14(0.98-1.3) | 1.16(0.96-1.3) | 1.1(0.8-1.4) | 1.05(0.85-1.3) |
| **Place of residence** |  |  |  |  |  |  |  |
| Rural | 1 | 1 | 1 | 1 | 1 | 1 | 1 |
| Urban | 1.8(1.3-2.3) | 0.89(0.52-1.51) | 0.96(0.5-1.7) | 1.2(1.04-1.5) | 1.14(0.92-1.4) | 1.2(0.95-1.6) | 0.91(0.7-1.18) |
| **Household size** |  |  |  |  |  |  |  |
| Small household | 1 | 1 | 1 | 1 | 1 | 1 | 1 |
| Medium household | 0.5(0.36-0.72) | 0.32(0.18-0.57) | 0.57(0.28-1.14) | 0.86(0.66-1.1) | 0.6(0.46-79) | 0.62(0.43-0.9) | 1.03(0.72-1.4) |
| Large household | 0.65(0.46-0.92) | 0.39(0.22-0.71) | 0.37(0.17-0.8) | 1.06(0.82-1.38) | 0.63(0.47-0.83) | 0.89(0.62-1.2) | 1.01(0.7-1.47) |
| **Antenatal visit** |  |  |  |  |  |  |  |
| No | 1 | 1 | 1 | 1 | 1 | 1 | 1 |
| Some visits | 1.9(1.38-2.5) | 1.03(0.6-1.7) | 0.96(0.52-1-76) | 1.6(1.3-2.03) | 1.4(1.14-1.86) | 1.05(0.79-1.3) | 1.8(1.4-2.5) |
| **Place of delivery** |  |  |  |  |  |  |  |
| Home & other | 1 | 1 | 1 | 1 | 1 | 1 | 1 |
| Public& private Health | 1.5(1.1-1.9) | 1.3(0.76-2.2) | 1.08(0.58-1.9) | 1.03(0.86-1.2) | 1.14(0.9-1.4) | 0.79(0.6-1.03) | 0.8(0.6-1.12) |
| **Working staus** |  |  |  |  |  |  |  |
| No | 1 | 1 | 1 | 1 | 1 | 1 | 1 |
| Yes | 1.1(0.7-1.4) | 0.6(0.26-1.4) | 1.1(0.53-2.4) | 1.3(1.06-1.6) | 1.06(0.8-1.43) | 1.05(0.79-1.49) | 1.5(1.14-2.03) |
| **Wealth index** |  |  |  |  |  |  |  |
| Poorest& porrer | 2.5(1.7-3.6) | 1.4(0.7-2.7) | 2.2(0.96-4.7) | 1.48(1.15-1.9) | 1.39(1.04-1.8) | 0.87(0.6-1.2) | 1.18(0.84-1.65) |
| Middle | 1.6(1.2-2.3) | 1.4(0.73-2.5) | 1.9(0.93-3.9) | 1.3(1-1.59) | 1.18(0.9-1.5) | 0.95(0.67-1.3) | 0.89(0.64-1.2) |
| Richer & Richest | 1 | 1 | 1 | 1 | 1 | 1 | 1 |
| **Mother’s Education** |  |  |  |  |  |  |  |
| No | 1 | 1 | 1 | 1 | 1 | 1 | 1 |
| Primary | 0.7(0.5-1.2) | 2.4(1.2-4.6) | 2.2(1.05-4.4) | 0.9(0.7-1.26) | 1.06(0.79-1.4) | 1.13(0.79-1.6) | 1.09(0.78-1.54) |
| Secondary& Higher | 0.8(0.5-1.07) | 1.5(0.85-2.9) | 2(1.18-5.7) | 0.8(0.72-1.08) | 0.93(0.72-1.19) | 1.1(0.74-1.38) | 1.04(0.78-1.4) |
| **Governorate** |  |  |  |  |  |  |  |
| Urban | 1.2(0.5-2.5) | 0.84(0.26-2.64) | 0.37(0.06-2.3) | 1.34(0.8-2.2) | 3(1.34-6.5) | 1.83(0.92-3.6) | 1.8(0.8-4.09) |
| Lower Egypt urban | 1.28(0.56-2.9) | 0.26(0.05-1.2) | 0.16(0.01-1.7) | 0.9(0.57-1.73) | 1.38(0.59-3.2) | 0.72(0.32-1.6) | 1.02(0.4-2.4) |
| Lower Egypt rural | 3.7(0.5-27.3) | 0.67(0.08-5.2) | 1.11(0.02-5.4) | 0.58(0.3-1.08) | 0.6(0.27-1.2) | 0.41(0.17-0.9) | 0.46(0.18-1.2) |
| Upper Egypt urban | 2.4(1.16-5.2) | 0.59(0.17-2.03) | 2.6(0.59-11.7) | 1.97(1.2-3.2) | 3.3(1.5-7.4) | 0.96(0.5-2) | 2.3(1.04-5.24) |
| Uper Egyot Rural | 6.9(0.96-50.5) | 1.8(0.24-13.9) | 0.09(0.02-0.4) | 1.4(0.7-2.6) | 1.3(0.65-2.8) | 1.04(0.4-2.4) | 2.06(0.8-5.2) |
| Frontier | 1 | 1 | 1 | 1 | 1 | 1 | 1 |
